# Omission of Postoperative Radiotherapy in Women Aged 65 Years or Older With Tubular Carcinoma of the Breast After Breast-Conserving Surgery

**DOI:** 10.3389/fonc.2018.00190

**Published:** 2018-05-30

**Authors:** San-Gang Wu, Wen-Wen Zhang, Jia-Yuan Sun, Feng-Yan Li, Yong-Xiong Chen, Zhen-Yu He

**Affiliations:** ^1^Department of Radiation Oncology, Xiamen Cancer Hospital, The First Affiliated Hospital of Xiamen University, Xiamen, China; ^2^Department of Radiation Oncology, Sun Yat-sen University Cancer Center, State Key Laboratory of Oncology in South China, Collaborative Innovation Center of Cancer Medicine, Guangzhou, China; ^3^Eye Institute of Xiamen University, Fujian Provincial Key Laboratory of Ophthalmology and Visual Science, Medical College, Xiamen University, Xiamen, China

**Keywords:** breast cancer, tubular carcinoma, elderly, breast-conserving surgery, radiotherapy

## Abstract

**Introduction:**

To investigate the temporal trends of postoperative radiotherapy (RT) administration and the effects of omitting postoperative RT on breast cancer-specific survival (BCSS) in women aged ≥65 years with tubular carcinoma (TC) of the breast who received breast-conserving surgery (BCS).

**Methods:**

We included women aged **≥**65 years with non-metastatic TC of the breast who underwent BCS between 2000 and 2013 using the Surveillance, Epidemiology, and End Results database. Statistical analyses were performed using chi-square tests, Kaplan–Meier analyses, Cox proportional hazards models, and a 1:1 propensity score matching (PSM).

**Results:**

Before PSM, a total of 1,475 patients with tumor size ≤2 cm, node-negative disease, and estrogen receptor-positive disease were identified, including 927 (62.8%) underwent postoperative RT and 548 (37.2%) had postoperative omission of RT. The administration of postoperative RT steadily declined over the study period. Patients with younger age, larger tumor size, and other race/ethnicity were more likely to receive postoperative RT. The median follow-up duration was 85.0 months, the 5- and 10-year BCSS rates were 98.7 and 97.9%, respectively. The median BCSS was 161.9 and 165.0 months for patients with and without postoperative RT, respectively, and the corresponding 5-year BCSS rates were 98.5 and 98.8%, respectively (*p* = 0.134). Prognostic analysis indicated that postoperative RT was not associated with improved BCSS rates compared with RT omission (*p* = 0.134). After PSM, a total of 431 complete pairs were generated. In the matched population, the 5-year BCSS rates were 98.6 and 98.4% in non-postoperative RT and postoperative RT groups, respectively (*p* = 0.858). The univariate analyses also confirmed that the administration of postoperative RT was not associated with better BCSS (*p* = 0.858).

**Conclusion:**

The incidence of breast cancer-related death is probably sufficiently low to avoid postoperative RT in women aged ≥65 years with TC of the breast after BCS.

## Introduction

Tubular carcinoma (TC)—a rare but distinct histological variant of well-differentiated invasive breast cancer—is characterized by the stromal invasion of well-formed tubular or glandular structures, and accounts for 1–2% of invasive breast cancer in the screening programs era (1–3). TC is generally associated with a better survival outcome compared with invasive ductal carcinoma, and is rarely known to form metastases (4). The better prognosis in TC of the breast may be related to the special clinicopathologic features, including small tumor size, node-negative, hormone receptor-positive, human epidermal growth factor receptor 2 (HER2)-negative, and lower tumor grade disease (1, 5–8). In addition, most of these patients received breast-conserving surgery (BCS) followed by postoperative radiotherapy (RT) (6, 7). Nevertheless, the role of postoperative RT in TC of the breast after BCS remains controversial, particularly among women aged ≥65 years (2, 6, 9, 10).

Breast-conserving surgery is the standard of care for the treatment of early-stage breast cancer (11) and has been shown to be equivalent to mastectomy in terms of survival outcomes (12). In patients aged ≥65 years with node-negative and hormone receptor-positive disease who underwent BCS, the omission of postoperative RT may increase the incidence of ipsilateral *breast* tumor recurrence (1.3 vs. 4.1%); however, the survival outcomes, including regional recurrence, distant metastases, and overall survival (OS) were comparable (13). Nevertheless, the effect of histological subtype stratification on survival outcomes remains unclear. In fact, this is the major unresolved aspect of the management of postoperative RT in elderly patients with TC of the breast after BCS. In addition, most of the previous studies included a limited number of patients or only using OS rather than breast cancer-specific survival (BCSS) as the survival endpoint (2, 6, 9, 10). In this study, we used a large population-based cancer registered database [Surveillance, Epidemiology, and End Results (SEER)] to investigate the temporal trends of postoperative RT administration and effects of omitting postoperative RT on BCSS in patients with TC of the breast after BCS.

## Patients and Methods

### Patients

We reviewed patients with TC of the breast from SEER program between 2000 and 2013. The SEER database is maintained by the National Cancer Institute, represents approximately 28% of the United States population, and provides accurate, timely, and continuous data of cancer incidence, patient demographics, and survival (14). The International Classification of Disease-0–3 code included in our study was 8,211/3. Patients who met the following inclusion criteria were included: (1) women aged ≥65 years with newly diagnosed non-metastatic TC of the breast; (2) had undergone BCS with or without postoperative beam RT; and (3) with available demographic, clinicopathologic, and treatment data, including age, race/ethnicity, grade, tumor stage, nodal stage, estrogen receptor (ER) status, progesterone receptor (PR) status, and receipt of chemotherapy. A total of 1,604 patients who met the study criteria, with 97.1, 96.0, and 98.3% of patients were tumor size ≤2 cm (T1 stage), node-negative disease, and ER positive disease, respectively. Therefore, we only included patients with T1 stage, node-negative disease, and ER positive disease in the finally analysis. Since SEER began recording the HER2 status after 2010, we only analyzed the HER2 data after 2010. BCSS was the primary endpoint of this study and was defined as the duration between diagnosis and death from breast cancer, or until censor at last contact. The ethics committee of the First Affiliated Hospital of Xiamen University approved this study.

### Statistical Analysis

The balance among the patient demographics, clinicopathological, and treatment variables was compared using chi-squared test or Fisher’s exact probability tests according to RT administration. Multivariable logistic regression modeling was performed to determine the predictive factors for RT administration. To reduce the effect of selection bias in the retrospective studies, a 1:1 match was performed using propensity score matching (PSM) method with following variables: age, race/ethnicity, tumor grade, tumor size, PR status, and receipt of chemotherapy (15, 16). Survival curves were plotted using the Kaplan–Meier method and then compared with the log-rank test. We calculated hazard ratios and their corresponding 95% confidence intervals (CIs) using Cox proportional hazards regression models to evaluate prognostic indicators related to BCSS. Back stepwise Cox multivariate analyses included variables that were statistically significant in the univariate analysis. All analyses were conducted using version 22 of the SPSS Statistical Software (IBM Corporation, Armonk, NY, USA) and the software STATA (Version 14.0; Stata Corp., College Station, TX, USA), and a *p* value of <0.05 was considered to indicate statistical significance.

## Results

**Figure 1:**
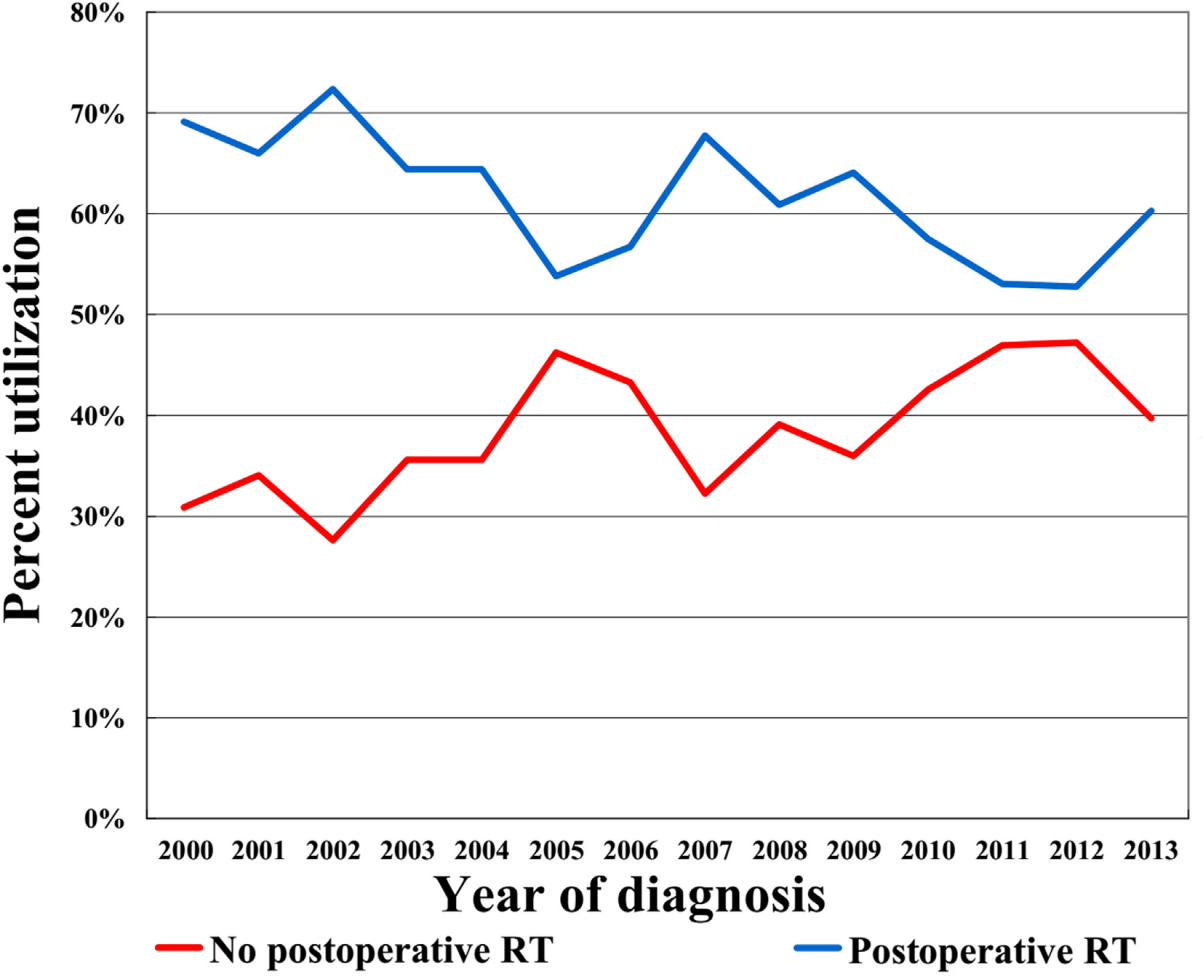
Utilization of postoperative radiotherapy vs. omission during the study period.

In the entire cohort, we identified 1,475 patients (median age, 73 years; range, 65–94 years), with 927 (62.8%) underwent postoperative RT and 548 (37.2%) did not. Figure 1 illustrates the temporal trends of postoperative RT administration from 2000 to 2013 and clearly shows that the RT administration rate steadily declined over the study period. Table 1 shows the demographic, clinicopathologic, and treatment variables before PSM. In most cases, the disease was well-differentiated, PR-positive, and HER2 negative. In addition, chemotherapy was not administered in most patients. After PSM, a total of 431 completely matched pairs were generated (Table 1).

**Table 1:** Patient characteristics before and after propensity score matching.

Variables	Before PSM				After PSM			
	*n*	No RT (%)	RT (%)	*p*	*n*	No RT	RT	*p*
**Age (years)**								
65–74	872	226 (41.2)	646 (69.7)	<0.001	444	222	222	1
75–84	484	227 (41.4)	257 (27.7)		372	186	186	
≥85	119	95 (17.3)	24 (2.6)		46	23	23	

**Race/ethnicity**								
Non-Hispanic White	1,307	489 (89.2)	818 (88.2)	0.064	782	391	391	1
Non-Hispanic Black	45	16 (29.1)	29 (3.1)		16	8	8	
Hispanic	60	28 (5.1)	32 (3.5)		38	19	19	
Other	63	15 (2.7)	48 (5.2)		26	13	13	

**Grade**								
Well differentiated	1,387	513 (93.6)	874 (94.3)	0.772	818	409	409	1
Moderately differentiated	81	32 (5.8)	49 (5.3)		44	22	22	
Poorly/undifferentiated	7	3 (0.5)	4 (0.4)					

**Tumor stage**								
T1mic	16	8 (1.5)	8 (0.9)	0.004	6	3	3	1
T1a	400	176 (32.1)	224 (24.2)		250	125	125	
T1b	711	240 (43.8)	471 (50.8)		408	204	204	
T1c	348	124 (22.6)	224 (24.2)		198	99	99	

**PR status**								
Negative	258	81 (14.8)	177 (19.1)	0.040	136	68	68	1
Positive	1,217	467 (85.2)	750 (80.9)		726	363	363	

**Her2 status (***n*** = 263)**								
Negative	257	114 (97.4)	143 (97.9)	1	–	–	–	–
Positive	6	3 (2.6)	3 (2.1)		–	–	–	

**Chemotherapy**								
No/unknown	1,447	542 (98.9)	905 (97.6)	0.113	852	426	426	1
Yes	28	6 (1.1)	22 (2.4)		10	5	5	

*“–” Indicates no data*.

*Her2, human epidermal growth factor receptor 2; RT, radiotherapy; T, tumor; PR, progesterone receptor; PSM, propensity score matching*.

Multivariable logistic regression analysis was performed to assess factors independently related to postoperative RT administration (Table 2), the results indicated that patients with younger age (<75 years), larger tumor size (T1b and T1c), and other race/ethnicity were more likely to receive postoperative RT. There was no association between postoperative RT administration and tumor grade, PR status and chemotherapy administration.

**Table 2:** Multivariable logistic regression analysis for predictive factors of postoperative radiotherapy administration before propensity score matching.

Variables	Odds ratio	95% CI	*p*
**Age (years)**			
65–74	1		
75–84	0.390	0.308–0.494	<0.001
≥85	0.088	0.054–0.142	<0.001

**Race/ethnicity**			
Non-Hispanic White	1		
Non-Hispanic Black	1.221	0.624–2.391	0.560
Hispanic	0.657	0.379–1.140	0.135
Other	2.026	1.091–3.765	0.025

**Grade**			
Well differentiated	1		
Moderately differentiated	0.850	0.517–1.397	0.521
Poorly/undifferentiated	0.605	0.129–2.842	0.524

**Tumor stage**			
T1a	1		
T1mic	0.840	0.300–2.346	0.739
T1b	1.573	1.203–2.056	0.001
T1c	1.501	1.095–2.059	0.012

**PR status**			
Negative	1		
Positive	0.745	0.549–1.012	0.060

**Chemotherapy**			
No/unknown	1		
Yes	1.610	0.634–4.090	0.316

*CI, confidence interval; T, tumor; PR, progesterone receptor*.

**Figure 2:**
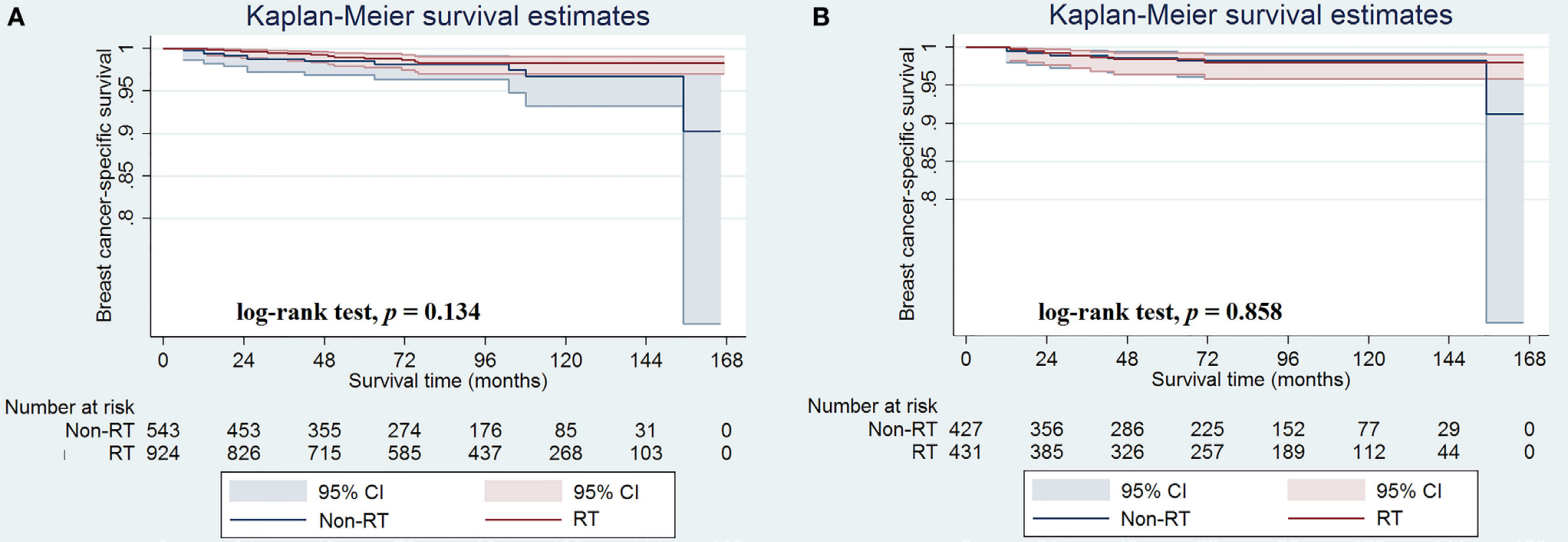
Breast cancer-specific survival rates in patients with and without postoperative radiotherapy before **(A)** and after **(B)** propensity score matching.

In the unmatched population, the median follow-up duration was 85.0 months (range, 0–167 months). A total of 421 patients died during the study period, including 23 who died from breast cancer-related disease. Overall, the 5- and 10-year BCSS rates were 98.7 and 97.9%, respectively, whereas the 5- and 10-year OS rates were 85.2 and 63.7%, respectively. The median BCSS was 161.9 and 165.0 months for patients with and without postoperative RT, respectively, and the corresponding 5-year BCSS rates were 98.5 and 98.8%, respectively (log-rank test, *p* = 0.134; Figure 2A). Only 12 and 11 patients with and without postoperative RT died of breast cancer-related disease, respectively.

The results of univariate and multivariate analyses are displayed in Table 3. Age at diagnosis and receipt of chemotherapy were the prognostic factors of BCSS in the univariate analysis. We then evaluated the independent predictors of BCSS using multivariate Cox proportional hazards models, and age ≥75 years and chemotherapy administration were found to be associated with poor BCSS (*p* < 0.05 for all). Of note, postoperative RT was not associated with better BCSS compared with RT omission in univariate analysis (*p* = 0.140).

**Table 3:** Univariate and multivariate prognostic analyses before propensity score matching.

Variables	Univariate			Multivariate		
	HR	95% CI	*p*	HR	95% CI	*p*
**Age (years)**						
65–74	1			1		
75–84	2.550	1.053–6.177	0.038	2.817	1.151–6.892	0.023
≥85	3.516	0.940–13.150	0.062	4.301	1.122–16.494	0.033

**Race/ethnicity**						
Non-Hispanic White	1			–		
Non-Hispanic Black	3.785	0.878–16.316	0.074	–	–	–
Hispanic	1.167	0.156–8.730	0.880	–	–	–
Other	1.119	0.150–8.364	0.913	–	–	–

**Grade**						
Well differentiated	1			–		
Moderately, poorly, and undifferentiated	0.612	0.082–4.544	0.631	–	–	–

**Tumor stage**						
T1a	1			–		
T1mic	–	–	0.982	–	–	–
T1b	0.760	0.264–2.191	0.611	–	–	–
T1c	1.825	0.649–5.127	0.254	–	–	–

**PR status**						
Negative	1			–		
Positive	2.510	0.588–10.707	0.214	–	–	–

**Chemotherapy**						
No/unknown	1			1		
Yes	7.554	2.242–25.452	0.001	9.857	2.855–34.035	<0.001

**Radiotherapy**						
No	1			–		
Yes	0.539	0.237–1.225	0.140	–	–	–

*“–” Indicates no data*.

*CI, confidence interval; HR, hazard ratio; T, tumor; PR, progesterone receptor*.

In the matched population, there were seven and seven patients in non-postoperative RT and postoperative RT groups died with breast cancer-related disease, respectively, and the 5-year BCSS rates were 98.6 and 98.4%, respectively (log-rank test, *p* = 0.858; Figure 2B). The univariate analyses also confirmed that age at diagnosis and chemotherapy administration were the prognostic factors of BCSS, while the administration of postoperative RT was not associated with better BCSS (HR 0.909, 95% CI 0.319–2.593, *p* = 0.858) (Table 4). We did not perform multivariate analysis because there were limited patients with breast cancer-related deaths.

**Table 4:** Univariate prognostic analyses after propensity score matching.

Variables	HR	95% CI	*p*
**Age (years)**			
65–74	1		
75–84	4.511	1.234–16.494	0.023
≥85	3.976	0.408–38.801	0.235

**Race/ethnicity**			
Non-Hispanic White	1		
Non-Hispanic Black	4.445	0.576–34.301	0.153
Hispanic	–	–	0.985
Other	–	–	0.989

**Grade**			
Well differentiated	1		
Moderately, poorly, and undifferentiated	1.170	0.152–8.994	0.880

**Tumor stage**			
T1a	1		
T1mic	–	–	0.983
T1b	0.517	0.138–1.934	0.327
T1c	1.402	0.404–4.864	0.594

**PR status**			
Negative	1		
Positive	2.685	0.351–20.528	0.341

**Chemotherapy**			
No/unknown	1		
Yes	14.486	3.198–65.623	0.001

**Radiotherapy**			
No	1		
Yes	0.909	0.319–2.593	0.858

*“–” Indicates no data*.

*CI, confidence interval; HR, hazard ratio; T, tumor; PR, progesterone receptor*.

## Discussion

Postoperative RT was not associated with better survival outcomes in certain lower risk-breast cancer patients aged ≥65 years after BCS (13). However, these results were obtained from a heterogeneous population without subtype stratification. In this study, we used a population-based registered database to investigate the role of postoperative RT for women aged ≥65 years with TC of the breast after BCS. Our results showed that postoperative RT was not a prognostic factor for BCSS, and that BCSS rates were similar between patients with and without postoperative RT in unmatched and matched populations.

Moreover, in this study, the 10-year BCSS rate was 97.9%, which was similar to previous studies (5, 17–19). Thus, patients with TC of the breast have excellent survival outcomes, and several studies have found that the survival of TC patients appears to be close to normal life expectancy (19, 20). In addition, only 1.9% of patients received chemotherapy in our study. These excellent survival outcomes support the adequacy of following a conservative approach to the treatment of TC, particularly in elderly patients.

The local recurrence rate is the major factor influencing the decision of adjuvant RT for TC of the breast. We conducted two literature reviews to determine whether postoperative RT can be avoided in patients with TC of the breast. One of the reviews included 736 patients with TC of the breast from 8 retrospective studies, and the local recurrence rates were 3.7% (22/593) and 9.8% (14/143) in patients with and without postoperative RT, respectively (21). These findings are consistent with the study by Sullivan et al., which included 11 retrospective series, and reported that postoperative RT administration was associated with significantly better local control as compared with RT omission (3.4 vs. 8.3%; *p* < 0.005) (2). Thus, postoperative RT administration has potential benefits of local control for patients with TC of the breast. However, the incidence of local recurrence in TC of the breast is extremely small, as compared with that in other histological subtypes (22).

Only a few studies have assessed the role of postoperative RT in elderly patients who receive BCS. Sullivan et al. found that, of 13 elderly patients who underwent BCS without any subsequent adjuvant RT (median age, 74 years), none developed local failure (2). In addition, the median time of local recurrence was more than 5 years (21). However, several studies acknowledged that it was difficult to distinguish between true recurrence and second primary breast cancer, or did not report the histological type of the local recurrence (2, 7, 9, 10, 17, 19, 23, 24). Hence, the heterogeneity in the TC data might make it difficult to accurately compare the results of local failure and may affect the judgment regarding the decision of postoperative RT in TC patients.

Accordingly, the role of postoperative RT in TC of the breast remains controversial. A previous SEER study included 6,465 patients with TC of the breast between 1992 and 2007; in that study, a total of 5,149 patients received BCS, and 70.4% of the patients were treated with postoperative RT. The study showed that adjuvant RT led to an almost 20% improvement in OS in patients aged >65 years. However, the OS benefits may likely be due to unaccounted patient selection bias, because most of the patients in this age group may have significant medical comorbidities. Therefore, physicians were more likely to omit adjuvant RT for those who were less healthy (6). A study including 248 TC of the breast patients from two German series indicated that the 10-year OS for patients in the RT group was significantly better (85.9 and 76.3% in the RT and non-RT groups, respectively; *p* = 0.035); however, the study only reported the survival endpoint of OS, but not BCSS (25). By contrast, a large study with 307 patients with TC of the breast failed to show a benefit in local control with postoperative RT after BCS (9). Another study by Sullivan et al. also found that additional RT following BCS was not significantly associated with better local control in elderly patients (2). However, in a study of by Hansen et al. included 115 patients, the 5-year relapse-free survival rate was 100 and 89% in patients with and without RT (*p* = 0.001); the median age of the non-RT group and RT group in that study was 55 and 54.5 years, respectively (21).

In this study, although patients with older age were less likely to receive postoperative RT, we found that postoperative RT did not improve BCSS in those patients. The heterogeneity of the study cohort in terms of age, centralized pathologic review, and histological inclusion criteria may be the main reasons for the differences in the abovementioned studies. As the life expectancy of cases of TC is close to normal (19, 20), it is important to balance the adverse effects and the survival effects for patients after BCS and postoperative RT. Our results support the decision that postoperative RT may be avoided in patients with TC of the breast after BCS (20).

Nevertheless, this study has certain limitations. First, there is inherent bias in a retrospective study. Second, two subtypes (pure type and mixed type) of TC of the breast have been described (26, 27); however, both types have similar clinical outcomes (2, 7, 23). In addition, the SEER database lacked information on centralized pathologic review, details of RT, chemotherapy and hormonal therapy, and patterns of local and distant failures. Moreover, the RT data had high specificity, although the overall sensitivity was 80% in the current SEER program (28).

## Conclusion

In conclusion, our results suggest that it may be safe to avoid postoperative RT in women aged ≥65 years with TC of the breast after BCS. Nevertheless, additional studies may be needed to adequately assess the role of postoperative RT in this special subtype of breast carcinoma.

## Ethics Statement

The study was approved by the ethics committee of the First Affiliated Hospital of Xiamen University.

## Author Contributions

S-GW, W-WZ, Y-XC, and Z-YH are lead authors who participated in data collection, manuscript drafting, table/figure creation, and manuscript revision. W-WZ, F-YL, and J-YS aided in data collection. S-GW and W-WZ are senior authors who aided in drafting the manuscript and manuscript revision. Y-XC and Z-YH are the corresponding authors who initially developed the concept and drafted and revised the manuscript. All the authors read and approved the final manuscript.

## Conflict of Interest Statement

The authors declare that the research was conducted in the absence of any commercial or financial relationships that could be construed as a potential conflict of interest.
